# Social relationships, mental health, and stigma: A qualitative study of social networks and HIV disclosure in Western Kenya

**DOI:** 10.1371/journal.pgph.0006273

**Published:** 2026-06-24

**Authors:** Sarah Obatsa, Adel Mburia-Mwalili, Salvador Jauregui, Karla D. Wagner, Lukoye Atwoli, Francesca Odhiambo, Jayne Lewis-Kulzer, Suzanne Goodrich, Kara Wools-Kaloustian, Jennifer L. Syvertsen

**Affiliations:** 1 Kenya Medical Research Institute (KEMRI), Kisumu, Kenya; 2 School of Public Health, University of Nevada, Reno, Nevada, United States of America; 3 Department of Anthropology, University of California, Riverside, California, United States of America; 4 Department of Mental Health and Behavioural Sciences, Moi University School of Medicine, Eldoret, Kenya; 5 Brain and Mind Institute, and Department of Medicine, Medical College East Africa, The Aga Khan University, Karachi, Pakistan; 6 Department of Obstetrics, Gynecology & Reproductive Sciences, Bixby Center for Global Reproductive Health, University of California, San Francisco, California, United States of America; 7 Department of Medicine, School of Medicine, Indiana University, Bloomington, Indianapolis, United States of America; Islamic Azad University South Tehran Branch, IRAN, ISLAMIC REPUBLIC OF

## Abstract

The disclosure of one’s HIV-positive status is a personal decision that can play an important role in engagement in HIV treatment and care. Social network approaches situate individuals within broader webs of social connections that influence health and wellbeing and thus hold the potential to elucidate key social factors shaping the process of disclosure. As part of a larger clinical study examining how substance use and mental health shape HIV outcomes, we recruited 61 individuals newly diagnosed with HIV at two clinics in Western Kenya to participate in an egocentric social networks study. We conducted a survey that generated a visual network map to guide a subsequent qualitative interview about experiences being diagnosed and living with HIV. We thematically analyzed the qualitative data and visual network maps to examine the social contexts and patterns of HIV disclosure, with the goal of identifying supportive contexts of disclosure. The mean age was 36.7 years (range: 20–62); women were significantly younger than men and more likely to self-report a mental health issue. Typically, participants disclosed their HIV-positive status to a small number of close, trusted alters in their network, including intimate partners, siblings and other family members, and friends. The need for mental health support in the wake of a new diagnosis, especially among women, encouraged disclosure. Across nearly all stories, stigma was a powerful deterrent to sharing one’s status, and multiple people noted prior disclosure to others not named in their current networks because their relationships were negatively impacted. Our study shows how the intersection of social relationships, mental health, and stigma is critical in understanding decision-making processes around disclosure. Selective disclosure typically improved participants’ wellbeing while stigma precluded disclosure and exacerbated distress. Our study offers suggestions for social network interventions to support people living with HIV/AIDS.

## Introduction

Disclosure of one’s HIV-positive status to social network members can play a critical role in facilitating engagement with HIV treatment, care, and support among people living with HIV/AIDS (PLWHA). Relationships based on trust and social support tend to facilitate disclosure in close relationships, which in turn can improve wellbeing [1,2]. Disclosure has also been linked to improved physical health outcomes, including encouraging health behaviors such as clinic attendance and medication adherence [3–5]. However, disclosure can also be a negative and psychologically distressing experience, particularly for women and vulnerable groups who may experience stigma, discrimination, violence, and other social repercussions due to their HIV-positive status [6–9]. Nondisclosure may be influenced by a range of psychosocial factors, including fear of rejection, previous adverse experiences related to disclosure, and concerns about imposing emotional or practical burdens on family members [10]. In particular, stigma is a powerful and well-documented barrier to disclosure [11,12]. Stigma can negatively impact mental health through inhibiting social support [13–15] and physical health due to missing medical appointments or not taking medication for fear of publicly outing oneself [16]. While social relationships can be positive and reinforce health behaviors and outcomes, the fears and anxiety associated with disclosing a life-changing diagnosis, especially within close relationships, are also critically important to understand.

Studying disclosure remains important in sub-Saharan Africa (SSA), where the global burden of HIV infection is concentrated and increased availability of antiretroviral medication has spotlighted disclosure as an important public health intervention to improve health outcomes and reduce transmission [17]. Across SSA, research suggests that disclosure is a complex process shaped by social, cultural, and interpersonal relationship factors [17–22]. As Madiba (2017) notes, stigma is a powerful threat to disclosure and “the availability and increased access to ART in SSA are not changing the underlying structural causes of stigma contributing to the reluctance of people to disclose” (page 107). As a form of shared knowledge about the discredited social status of HIV/AIDS, stigma toward PLWHA can operate through personal relationships and is often upheld by institutions and in cultural imaginations equating the disease with death, regardless of advancements in care [17]. Put another way, disclosure in SSA must be considered beyond the context of public health intervention to consider the social worlds that PLWHA navigate.

Our social network study draws on the disclosure process model to guide our thinking on when and why people navigate disclosure of their HIV status [23]. In conceptualizing disclosure not as a discrete event but as an integrated social process, the model considers the antecedents to disclosure, or how people calculate the social cost-benefits of disclosing; the disclosure event itself; and how the disclosing individual is impacted afterwards in terms of their psychological health and wellbeing, social support, and ability to manage a diagnosis that is now socially known by others. The model also builds in a feedback loop in terms of how the disclosure process will affect one’s openness about their status and willingness to disclose to others in the future. We situate this model within the broader social and cultural context of Kenya, where despite improvements in the HIV care cascade, an estimated 1.4 million people are living with HIV and the overall HIV prevalence of 3.3%, which is higher among females (4.5%) compared to males (2.2%), and varies across geographic regions [24]. In Kenya, stigma remains a key challenge in terms of HIV disclose [25–27], but research suggests that supportive social relationships can counter stigma and facilitate dialogue [28–30].

Taken together, disclosure of one’s HIV status is a deeply personal process that reflects and reshapes the social contexts of our lives. Social network approaches offer a valuable framework for elucidating the complex social and relational dynamics that shape decisions about how and why people disclose their HIV-positive status. Social network data collection and analysis conceptualize individuals as enmeshed in broader webs of social connections that influence health and wellbeing. This approach to data collection measures aspects of social connections and how relationships may directly shape health. Social network-informed intervention approaches have supported individuals in navigating a new HIV diagnosis and promoting intervention uptake in under-resourced communities [25,31–33]. In the current study, we use a qualitative social network approach to examine the social contexts and patterns of HIV disclosure among participants newly enrolled in HIV care to understand the experiences of sharing a life-altering diagnosis. Our overarching goal is to identify supportive contexts of HIV disclosure within participants’ social networks to confront stigma and nurture the health and wellbeing of PLWHA.

## Materials and methods

### Ethics statement

The research was approved by the Institutional Review Boards or Ethics Committees of the participating sites, including Moi University College of Health Sciences and Moi Teaching and Referral Hospital Institutional Research and Ethics Committee and the Kenya Medical Research Institute/Scientific Ethics and Review Unit. In the United States, Institutional Review Boards at Indiana University and the University of California, Riverside also reviewed all protocols. All individuals in this study provided written informed consent prior to study participation.

### Study setting

Participants were recruited from clinics in the East African region of the International Epidemiological Databases to Evaluate AIDS (EA-IeDEA) consortium. EA-IeDEA was established by the National Institute of Allergy and Infectious Diseases (NIAID) in 2005 to facilitate the collection and analysis of large cohorts of HIV/AIDS-related data and is one of seven regional networks in the global IeDEA consortium [34]. Participants in this study were enrolled from two IeDEA-affiliated programs: the *Academic Model* *Providing Access to Healthcare (AMPATH)* located in Eldoret, Kenya, and the *Lumumba Sub-county Hospital* located in Kisumu, Kenya. The AMPATH clinic is in Uasin Gishu County in western Kenya, where HIV prevalence is 5.5%. Lumumba is located in Kisumu County, which has one of the highest HIV prevalence rates in Kenya at 17.5%. AMPATH has approximately 88,000 patients active in HIV care and Lumumba serves 9,800 patients.

### The “Syndemics” parent study

The *“Syndemics*” cohort study examined the interlinked effects of alcohol and drug use and mental health on HIV outcomes among newly diagnosed adults in Kenya and Uganda. From January through October 2019, the S*yndemics* study recruited 576 PLWHA from three clinical sites, including 200 from AMPATH and 200 from Lumumba. Eligibility for *Syndemics* included being at least 18 years old and not previously enrolled into HIV care.

As part of a longer survey, *Syndemics* participants completed the Client Diagnostic Questionnaire (CDQ), which is a tool adapted from the Patient Health Questionnaire and based on DSM IV diagnostic criteria for identifying mental health and substance use disorders [35]. The CDQ is designed for use with PLWHA and to be administered by lay personnel with no formal mental health or substance use training. We translated and validated a version of the CDQ, which was available in English, Swahili, and Dholuo, based on participant preference [36]. Briefly, the CDQ has eight modules to evaluate self-reported symptoms of major depressive disorder, other depressive disorder, panic disorder, generalized anxiety disorder, PTSD, psychosis, alcohol dependence, and drug dependence. Each module starts with one or two initial screening questions asking about the presence and timeframe of general symptoms of each condition. If the participant answered ‘Yes’ to the initial screening questions, additional questions ascertained further information about the condition. A ‘No’ response to initial screening questions allowed the interviewer to skip to the next module. The standard scoring criteria for the CDQ was used to define a positive screen for each condition in the past six months. The CDQ scores helped guide our purposive sampling for the qualitative interviews in the next phase of the research, the networks sub-study described below.

### The social networks sub-study

The *Networks* sub-study sample was recruited from the *Syndemics* cohort study. Eligibility was open to anyone enrolled in the *Syndemics* cohort study in the two Kenyan sites. Between November 1, 2019, and March 31, 2020, the *Networks* study enrolled 174 people (88 from Lumumba in Kisumu; 86 from AMPATH in Eldoret). In March 2020, the research team ended recruitment for the *Networks* study due to the global COVID-19 pandemic and concerns for staff and participant safety.

All networks participants engaged in a brief survey and quantitative social networks questions within 12 months of enrolling in HIV care. RedCap was used for survey questions and Vennmaker software was used for social networks data collection. Of the 174 total *Networks* participants, 61 (n = 29 in Kisumu; n = 32 in Eldoret) were purposely sampled to participate in an additional qualitative interview immediately after the quantitative data collection. The 61 qualitative network participants comprise the sample for this analysis.

### Network quantitative data collection

Participants (“egos”) were asked to name and describe the characteristics of 10 of their social network members (hereafter: “alters”). A single name generator elicited the names of 10 people with whom the participants had communicated with (phone, email, social media, text, SMS, or in person) in the past three months. Name interpreters included role relations (e.g., intimate partner, family members), social support, trust, and conflict with network members. Alter-level measures included ego’s reports about their demographic information, including functional roles (e.g., partner, family, friend). Types of support elicited by each alter included material, emotional, health-related, mental health, leisure, and spiritual support. We also asked if egos disclosed their HIV status (yes/no) to each alter.

### Social network mapping

Vennmaker software generated social network maps based on participant responses in the egocentric network survey. We used a hierarchical mapping technique, in which participants were presented with three concentric circles, locating them (the “ego”) in the center. We asked participants to place their alters in the circles, based on their level of perceived emotional closeness. Using the egocentric survey data, each alter was displayed as a dot on the map and color coded to show disclosure (purple indicated they had been disclosed to; green indicated they had not). Around the dot, up to six types of support provided by that alter were displayed (i.e., emotional, mental health), based on the ego’s self-report. These network visualization maps were used to guide the qualitative interviews with the 61 participants.

### Network qualitative data collection

In addition to quantifying patterns of disclosure, we wanted to learn more about the social processes and experiences of HIV disclosure. Qualitative studies are well-suited to unpack the complex meanings embedded in disclosure for both PLWHA and those in their social worlds. Using the network maps to prompt reflection, open-ended questions asked about participants’ networks, including what motivated participants to disclose, who they had/had not disclosed to and why, and reactions and changes in relationships after disclosure. Interviews were conducted by trained Kenyan Research Assistants (RAs) from the community who had prior training and experience conducting surveys and qualitative interviews. Interviews lasted up to one hour.

We selected our qualitative sample for variation in sex, age, mental health status, and substance use (based on the CDQ screening previously described), factors theorized to affect experiences of HIV diagnosis and disclosure. The RAs also helped guide recruitment in terms of selecting participants who were open to sharing and with whom they had developed rapport. As the RAs were trusted members of the community with whom participants had already interacted in the parent *Syndemics* study, nearly everyone who was approached agreed to enroll in the *Network*s study; the five who declined did so due to time constraints.

We conducted interviews until we reached saturation of categories, meaning we achieved robust representation across our conceptual categories and thus felt confident we had interviewed a balanced cross-section of the larger *Networks* sample to account for a diversity of experiences. Based on their knowledge and experience, the RAs also helped determine the sample cutoff when they reported hearing common patterns across the interviews [37]. Interviews were audio recorded, transcribed, and translated for analysis in dual languages by the trained bi/trilingual interviewers. All transcripts retained both the original interview language and English translations (for interviews in Swahili and Dholuo) so that questions around language and meaning could be discussed among the research team as needed.

### Data analysis and integration

Descriptive statistics were calculated from the survey data. The qualitative data were coded by the team of trained Kenyan RAs who conducted the interviews, under supervision of the PI. The lead analyst of this study include the first author, a Kenyan RA (initials) who coordinated this study and has extensive experience with data collection. The PI (initials), who has extensive experience with qualitative research, supervised all study activities, including double checking the coding. The collaborative analysis process began by reading through the interviews and independently generating an initial list of codes based on the primary areas of interest and emergent themes [38]. We used deductive and inductive approaches, with deductive codes drawn from our research questions (e.g., why people chose to disclose) and inductive codes emerging organically from the data. We met to discuss and refine the codes and constructed a draft codebook for an initial round of coding. Codes were arranged in a hierarchical structure by parent codes (e.g., based on major themes) and corresponding sub-codes under each theme. The team held regular meetings to address questions, discrepancies, and refine the codes as needed until coding was completed. We used MAXQDA software to code and manage the data.

We used a constant comparison method to iteratively toggle between the qualitative data and network maps to assess patterns in the amount of disclosure within networks and determine the factors facilitating or prohibiting disclosure. The lead author identified the primary thematic categories to examine in this paper falling under the umbrella code of ‘disclosure,’ while the PI also read through these codes and took notes to corroborate the major themes. Across participant stories, relationship quality, including trust and support, mental health, and stigma consistently emerged as key factors shaping disclosure. After these themes were established, (initials) examined the network maps for visual patterns of disclosure. Specifically, we assessed the amount of disclosure in the network maps in terms of 1) the number of alters disclosed to; 2) closeness of alter relationships (as shown by the proximity of where each alter is placed in the concentric circles) and 3) the forms of support each alter provided (as depicted in the slivers of color inside the circles) in order to determine visual patterns. We looked for variation in patterns, selected examples, and returned to those participants’ full interview transcripts to read their stories. During this process, we realized that multiple participants also talked about people who were missing from their current network maps due to prior disclosure and their relationships changing. Collectively, we discussed a selection of cases, and the PI made a final determination of eight cases whose rich interviews best illustrated the primary patterns of disclosure. Based on the full interview texts, we wrote up holistic descriptions of participant disclosure experiences. Post-pandemic, we shared a preliminary version of our analysis in community dissemination events, which generated positive feedback, and supports the analysis presented here.

While we primarily focus on the qualitative interview data, we also draw from the survey data to describe the sociodemographics of the 61 participants and share their network maps as visual guides. Our results are organized by the key themes we identified across the qualitative interviews; the cases selected to represent each theme illustrate the complexities embedded in the disclosure process, including when and why people choose to disclose (or conceal) their diagnosis to the alters in their network. Our analysis describes the key contexts of disclosure and features direct quotes to highlight the voices of participants and show how varying levels of disclosure are shaped by complex social dynamics, trust, the need for support, and stigma. All names are pseudonyms.

## Results

### Socio-demographic characteristics

Table 1 draws from our survey data to illustrate the sociodemographic characteristics of the 61 participants, stratified by sex. The mean age was 36.7 years (range: 20–62), and women (34.5 years) were significantly younger than men (39.4 years; p = 0.0152). Educational attainment and marital status did not differ. Women were more likely to report mental health issues compared to men (71.9% vs 37.9%, p = 0.0077), including depression: (71.9% vs 17.2%, p < 0.0001), anxiety (46.9% vs 17.2%, p = 0.0138), and PTSD (40.6% vs 17.2%, p = 0.0455). Men were more likely to report alcohol (51.7% vs 12.5%, p = 0.0019) and drug use (11.5% vs 0%, p = 0.0455) compared to women. Participants disclosed to an average of 3.9 alters, ranging from zero to all 10 alters listed in their networks.

**Table 1:** Sociodemographic characteristics of participants (egos), stratified by gender (n = 61).

Characteristic	Total (n = 61)	Women (n = 32)	Men (n = 29)	p-value
Age (mean, sd, range)	36.7, 9.1 (20-62)	34.5, 9.0 (20-52)	39.4, 8.4 (24-62)	
Age-groups*				***0.0152***
18-24 years	7 (11.5)	6 (18.7%)	1 (3.5%)	
25-34 years	20 (32.8)	14 (43.8%)	6 (20.7%)	
35-44 years	18 (29.5)	5 (15.6%)	13 (44.8%)	
45 + years	16 (26.2)	7 (21.9%)	9 (31.0%)	
Education*				0.6819
None	1 (1.6)	1 (3.1%)	0 (0.0%)	
Primary	20 (32.8)	8 (25.0%)	12 (41.4%)	
High school	13 (21.3)	8 (25.0%)	5 (17.2%)	
Vocational/college	20 (32.8)	11 (34.4%)	9 (31.0%)	
University	7 (11.5)	4 (12.5%)	3 (10.3%)	
Marital status*				0.0956
Single	11 (18.0)	8 (25.0%)	3 (10.3%)	
Married	32 (52.5)	14 (43.8%)	18 (62.1%)	
Separated/divorced	7 (11.5)	6 (18.7%)	1 (3.5%)	
Widowed	6 (9.8)	3 (9.4%)	3 (10.3%)	
Common law	5 (8.2)	1 (3.1%)	4 (13.8%)	
Ethnicity*				0.1688
Luo	29 (48.3)	12 (37.5%)	17 (60.7%)	
Kalenjin	10 (16.7)	5 (15.6)	5 (17.9)	
Luhya	12 (20.0)	7 (21.9%)	5 (17.9%)	
Kikuyu	4 (6.7)	4 (12.5%)	0 (0.0)	
Other (Kisii)	5 (8.3)	4 (12.5%)	1 (3.6%)	
**Site**				0.5334
AMPATH (Eldoret)	32 (52.5)	18 (56.3%)	14 (48.3%)	
FACES (Kisumu)	29 (47.5)	14 (43.7%)	15 (51.7%)	
**Mental health issues**				
Any mental health	34 (55.7)	23 (71.9%)	11 (37.9%)	***0.0077***
Depression	28 (45.9)	23 (71.9%)	5 (17.2%)	***<.0001***
Anxiety	20 (32.8)	15 (46.9%)	5 (17.2%)	***0.0138***
PTSD	18 (29.5)	13 (40.6%)	5 (17.2%)	***0.0455***
Substance use*				
Alcohol	19 (31.2)	4 (12.5%)	15 (51.7%)	***0.0019***
Drugs	4 (6.6)	0 (0.0%)	4 (11.5%)	0.0455

* Fisher’s exact test.

### Qualitative themes of disclosure

Qualitatively, three major themes emerged. First, the quality of the participants’ (egos) social relationships were important: participants primarily disclosed to trusted network members who provided support, including intimate partners, immediate family members, children, and close friends. Trust and closeness were key drivers of disclosure; however, sometimes participants did *not* disclose to close people because of concerns that disclosure could threaten an important relationship or put their support at risk. Second, there is a complex relationship between disclosure, mental health and need for support, and the stigma that continues to surround HIV, including the anticipation of being treated differently after sharing a diagnosis. Finally, the people missing (i.e., not listed) in the ego’s networks lend insight into how social relations can change in the wake of sharing a diagnosis and influence future decisions to disclose to others. Below, we selected illustrative examples of each of these interconnected themes in our data to show the social complexity of the disclosure process through a holistic and participant-centered presentation of our results.

### Disclosure in close, trusted relationships

Most commonly, participants disclosed to a selective group of close, trusted alters. Disclosure was often motivated by needing support, and for many participants, disclosure clearly mapped onto their supportive network members. Participants also disclosed to close network members for practical reasons, including their inability to hide their medication or physical health symptoms. Disclosure was often facilitated when alters were themselves living with HIV or knew others with HIV and were more likely to understand and support participants in their new diagnosis.

James is a 31-year-old married man who has also only disclosed to his wife, “as the most important person that you have in your life is your wife.” After he tested positive, he knew he had to tell her because they are close, and he wanted to plan for their future together:

*I decided to tell her because of our life together, the future…because you know, if you have to have a future then you have to also find a way of planning. Then I also decided to do it so that it’s not good to harm her as well. You know for you to have healthy children, children who are free from HIV, a partner has to know.*

**Fig 1:**
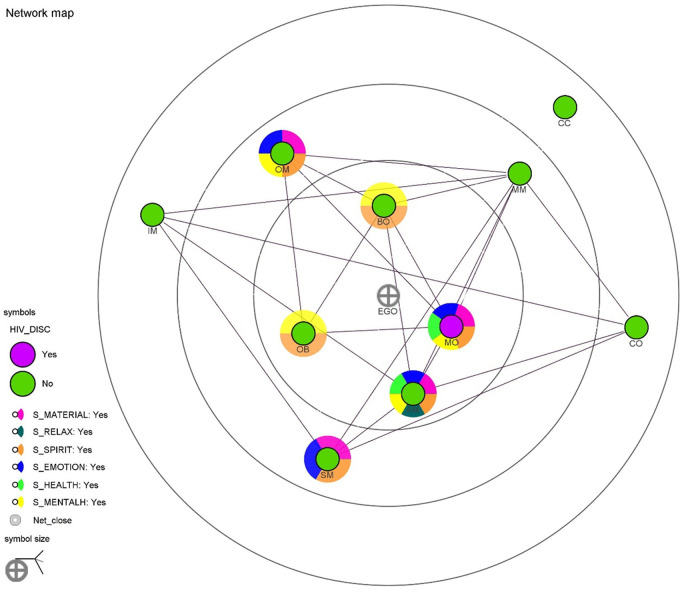
James’s network map. James has only disclosed to his wife because she is the most important person in his life. His wife has accepted his HIV diagnosis with understanding and has remained his strongest source of encouragement and stability. James maintains good physical and mental health, adheres consistently to treatment despite mild side effects like headaches and vivid dreams, and relies on emotional and practical support from his wife. He hopes to gradually strengthen his broader social connections through improved interaction and openness.

However, such conversations are difficult as they raise issues around trust and safety. His disclosure strategy was to take her to a different HIV facility from where he had tested, telling her, “You know it’s high time we need to know our status.” She tested negative; he tested positive again, and it forced a conversation. Even though she was initially confused and surprised by the diagnosis, his wife continued to offer critical emotional support. She said, “Well, maybe God knows why it happened.” As shown in Fig 1, James has not disclosed to others with whom he is not that close and from whom he does not receive support. He has not disclosed to people who he thought would ask too many questions, including his family and friends who are also in his inner circle and provide support. Paradoxically, James, like others in our study, did not disclose to some of his close relationships so as not to risk the support he needed or jeopardize those trusting relationships.

**Fig 2:**
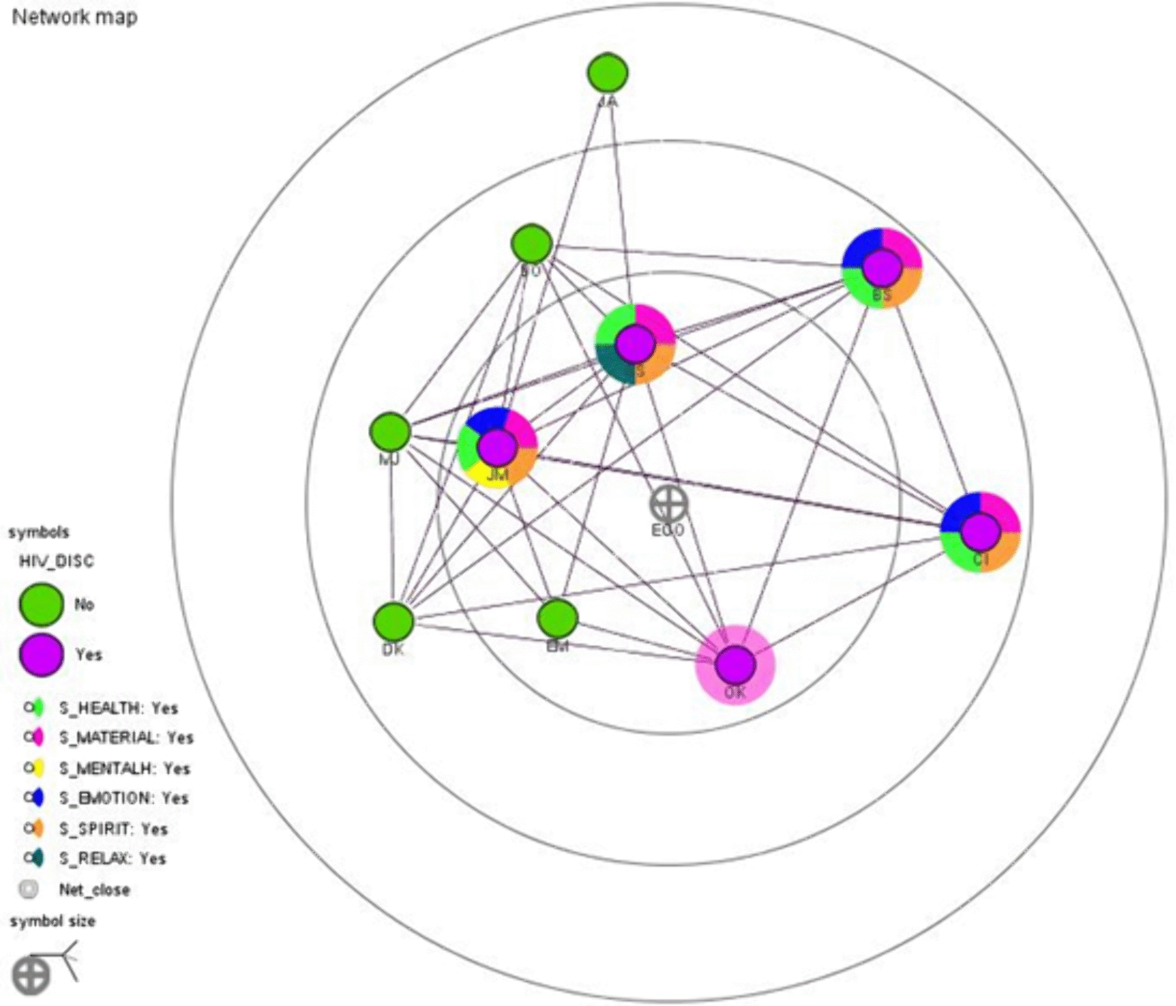
Ruth’s network map. Ruth’s map depicts that she disclosed to half of her network, all of whom provide support, versus no disclosure to those who do not provide support (the green dots). She has not disclosed her status to more distant relatives or acquaintances due to trust issues, fear of discrimination or gossip, and concern that such people would distance themselves, explaining that she sees no need to tell them and does not plan to disclose in the future. Instead, she relies on her supportive alters in managing her HIV, daily wellbeing, and emotional stability.

The role of support in disclosure is important and striking in Ruth’s network map (Fig 2). Ruth is a 30-year-old woman who has disclosed to her husband, sister, and three other people close to her. While some were “shocked,” the people to whom she disclosed provide her with many different forms of support, including spiritual support and leisure and relaxation, which helped reduce her stress and anxiety in managing her new HIV status. When asked about her decisions to disclose, support was key:

*I felt that they should know about my status just in case of anything. Be it accidents or any physical things, they can be able to know how to handle it. And some for spiritual support and emotional support, advice so that I cannot feel alone.*

Ruth called her sister her “prayer warrior” who prays with her and provides emotional support; other friends in her inner circle are key in helping her manage her depression, and offering encouragement like, “You are always the best. So, just be calm and everything will be okay.” In contrast, she has not disclosed to those in her outer circles who do not provide any support. She did not trust these alters as much, as they are neighbors and acquaintances whom she feared might stigmatize her if they knew their status. Thus, she felt no compelling need to disclose.

**Fig 3:**
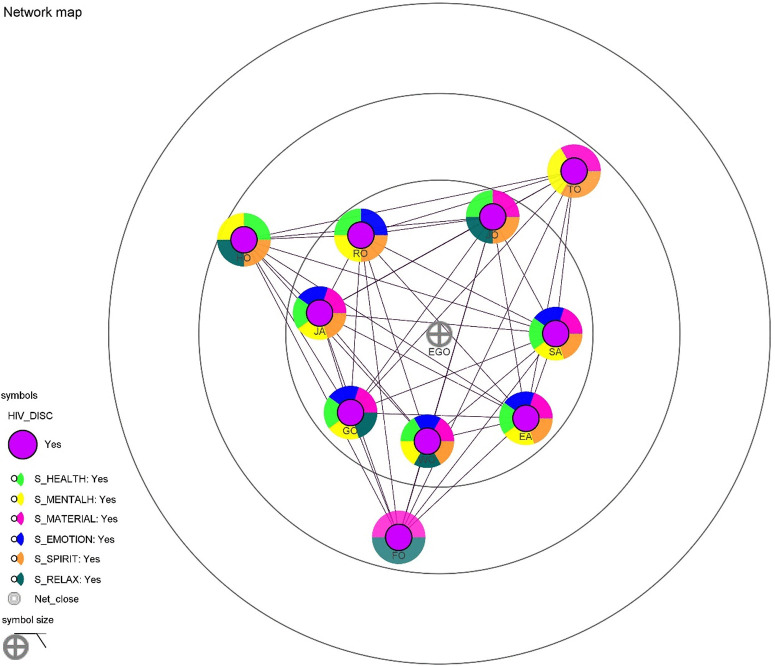
Victor’s network map. Victor disclosed to everyone in his network, all of whom provide different types of support. Overall, he maintains good physical and emotional health, adheres to his medication, and appreciates the spiritual and informational support, financial stability, strong faith, and trusted encouragement as the key forms of support he needs to remain healthy.

Victor is a 37-year-old widowed man; he was one of the few in the entire sample to disclose to everyone in his social network (Fig 3). Disclosing to his close and supportive network of family and friends has helped him navigate his diagnosis:

*There is one reason why I decided to tell them: keeping it to myself would have harmed me. I would have been stressed. That’s why I decided to share with my friends and my family.*

Immediately after his diagnosis, he made individual appointments with each person in his network, so he could share his diagnosis privately. He said everyone took it well, and his relationships have not changed, as they continue to support him and encourage him to keep his medical appointments and take his medication.

### Mental health, support, and stigma

Many participants, particularly women, discussed how their mental health status in the wake of their diagnosis motivated them to reach out for support, which required disclosure of their diagnosis. Often, individuals needed acute crisis support in the immediate aftermath of a diagnosis as well as longer-term support to cope and emotionally navigate their new status. Women talked about how their mental health shaped their decisions; disclosure was typically helpful while a lack of disclosure could exacerbate mental health concerns. Relatedly, the need for spiritual support was also a salient theme, as many people sought prayers, attended church, and sometimes asked pastors for guidance, which supported participants’ mental health and wellbeing.

**Fig 4:**
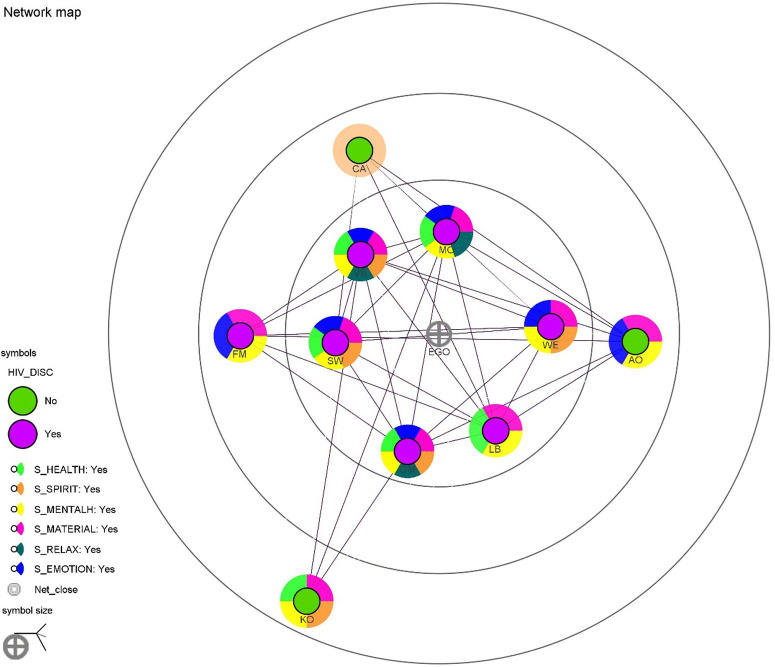
Rose’s network map. Rose has disclosed to most of her network, who have provided support in the wake of her diagnosis. She disclosed her HIV status to trusted loved ones because she was overwhelmed with depression after her diagnosis, explaining that these confidants reacted with reassurance and strengthened their relationship with her, whereas she avoids telling the alters on the outer edges of her network map due to fears of gossip, stigma, and potential distancing.

Rose is a 24-year-old married woman who disclosed her HIV status to nearly everyone in her network, including her husband, family, and friends (Fig 4). She quickly disclosed after her diagnosis because of her mental health:

*I decided to tell them because the first day I found out I had HIV, I was so depressed. Though I could pretend I was okay psychologically but inside me I was dying. So, I went to the house and sat down. And I started thinking, “if I keep this thing to myself, wouldn’t people question what’s wrong with me these days?” So, I had to express my feelings to them so that they could also give me advice. There are even those who told me, “You know what, we have it [HIV] too.” …The depression I had was what made me tell them.*

Rose receives ample support from her network and after disclosing her status, her relationships have “become stronger.” Importantly, her disclosure prompted others in her network to also disclose their HIV status to her in solidarity.

Rose is not as close to three of her friends placed in the outer circles of her network map. She has not disclosed to them because she feared being gossiped about: “They talk too much. They will start spreading my name outside there. They are not as quiet as the other ones I have told.” She said she needs to build up the “morale” to tell them, so they don’t find out accidentally or when she is obviously sick. However, she worried that the stigma of HIV would change her relationships.

Building off the stories of Rose and Ruth above, stigma emerged as common concern in the context of disclosure; in fact, stigma was interwoven across nearly every story of disclosure and was often related to mental health. Anticipated stigma manifested as the fear of being mistreated, discriminated against, shunned, or losing support; internalized stigma felt like such mistreatment was deserved and participants needed to carry the burden of a diagnosis on their own; and community stigma was raised in terms of how the broader community thought about HIV and accepted people with a diagnosis. Even among participants with high levels of network disclosure, stigma emerged as a key barrier to sharing, sometimes even in close relationships.

**Fig 5:**
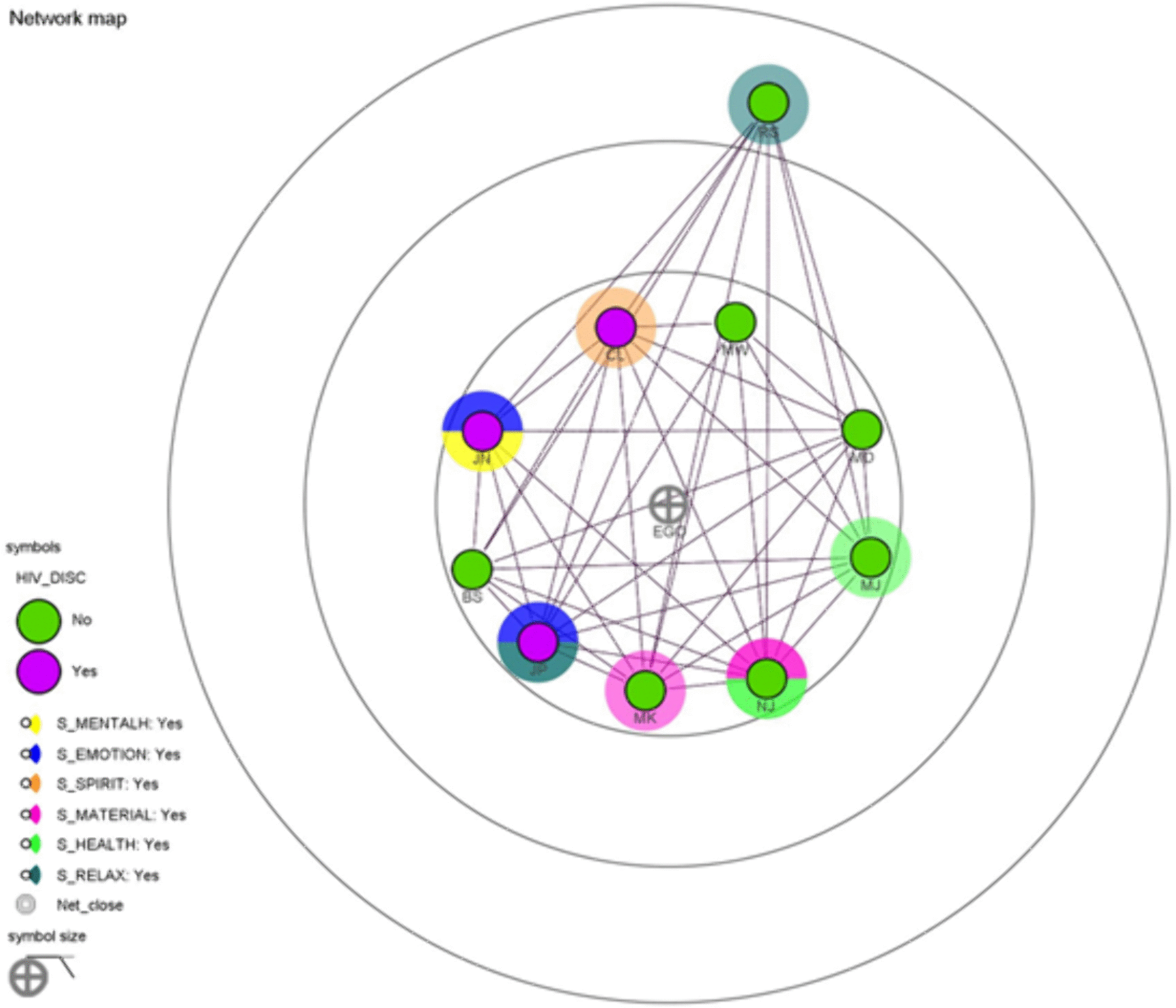
Esther’s network map. Esther has only disclosed to three people who are close to her for their support; she worries about how others will treat her. These close friends and her sister encourage her emotionally, remind her to take medication, and provide food or financial help. They also played a major role in restoring her hope when she initially felt suicidal after her HIV diagnosis. She has not disclosed her status to most people in her network due to fears of stigma, gossip, shock, or even violence, noting that some individuals might react negatively or distance themselves.

Esther is a 24-year-old married woman who has disclosed to two close friends and her sister, who are all “good people” (Fig 5). Her disclosure to her sister was driven by suicidal ideation, and the support she received has helped her accept her diagnosis. However, all three of these people advised her *not* to share her status more widely because of the harm it could cause:

*[My friend] was the first person to disclose to after I came from the test, she told me to just relax and that I don’t disclose to people and start taking my medication. I also told my sister and told her also that I wanted to commit suicide because I was as good as a dead person. My sister encouraged me and told me that there are so many people with HIV, they walk, eat and live well, the only thing I need to do is to take my medication. [My other friend] also told me the same, he also told me not to tell people but to stay strong…I think they knew because if you disclose to people, some will be happy, some will laugh at you, others will insult you, that is the reason why they told me to keep quiet and not to take it as something difficult, they will assist me wherever possible.*

However, not disclosing more widely to others in her network has affected Esther’s mental health and created internalized stigma. She has not disclosed to people who provide little or no support, including the friend who is placed in the outermost circle of her map, whom she fears somehow found out about her status and now “hates” her. She has not disclosed to other close friends because “I feel like they will hate me or they will distance themselves from me … I begin to pity myself and begin to think and see myself as not a human being.” Esther also fears other family members’ reactions, worrying they will be “shocked” and blame her for her condition. Thus, she relies on her small group of trusted, supportive alters to help her navigate her diagnosis.

**Fig 6:**
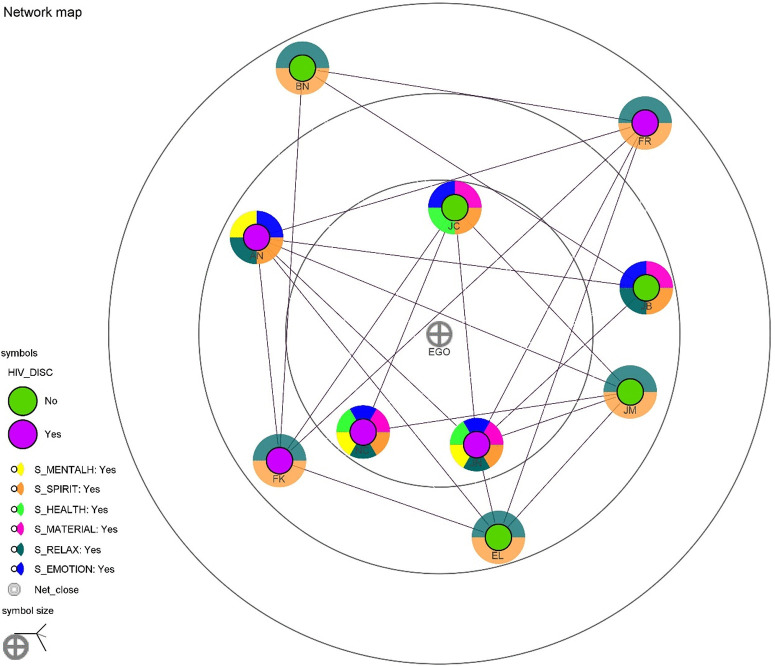
Enoch’s network map. Enoch has a supportive network; they have disclosed to selective family members and friends who have helped support them through their diagnosis and overcome self-stigma. Enoch maintains good physical and mental health through medication adherence, acceptance, supportive relationships, reduced alcohol use, and a positive outlook.

In contrast, Enoch’s story illustrates the importance of close relationships in providing support and combatting the stigma of a new diagnosis. Enoch is a 30-year-old man who identifies with the LGBTQIA+ community. They realized their sexual orientation early on; their sister helped them tell their parents, who were furious at first, but with time and counselling have accepted Enoch. Disclosure has been a process for Enoch, and they had disclosed to about half of their network at the time of the interview (Fig 6). They receive a lot of support from family and friends regarding “financial problems, my sexual orientation, my HIV status, family issues….” Even so, Enoch has not disclosed to some of their network because of not knowing how they will react. For example, Enoch has also not disclosed to their brother, even though the brother has seen the medication in their kitchen cabinet. Enoch would prefer to wait until they directly asked about it.

Like Rose’s story above, several of Enoch’s friends are also HIV + , and having multiple people in their network who also shared their HIV status and accepted their sexuality has helped Enoch come to terms with their own diagnosis. The level of support within these relationships has not changed, and Enoch has been able to resist internalized stigma:

*[M]ost of them are [HIV] positive. How can you discriminate against your friend because he is positive, why are you not discriminating against yourself? …self-stigma begins from you as an individual … I’ve never been discriminated against by them.*

With the support of their network, Enoch adheres to their medication, reduced their alcohol consumption, and has found ways to emotionally cope. Their sister helped them reframe their diagnosis to appreciate that it is better to be tested and know their HIV status so they can stay healthy and persevere, rather than not know and live in denial. According to Enoch:

*You have to accept yourself, sexual orientation, first of all, you have to accept yourself with your situation… I said, ‘Thank God I’m HIV positive, I’m grateful for this. Let me just take my medicine… I’m always grateful for each and every situation…you have to look for a way of convincing yourself so that you move on with life.*

### Missing network members

Stigma shaped who was included and who was left out of network maps, as relationships sometimes changed in the wake of disclosure. Multiple participants who had disclosed to others in their networks reported that these relationships fell apart when they faced negative reactions to their HIV status; consequently, those people were not listed in their current network maps. Negative experiences led participants to be cautious in further disclosing their status, which often negatively impacted their mental health.

**Fig 7:**
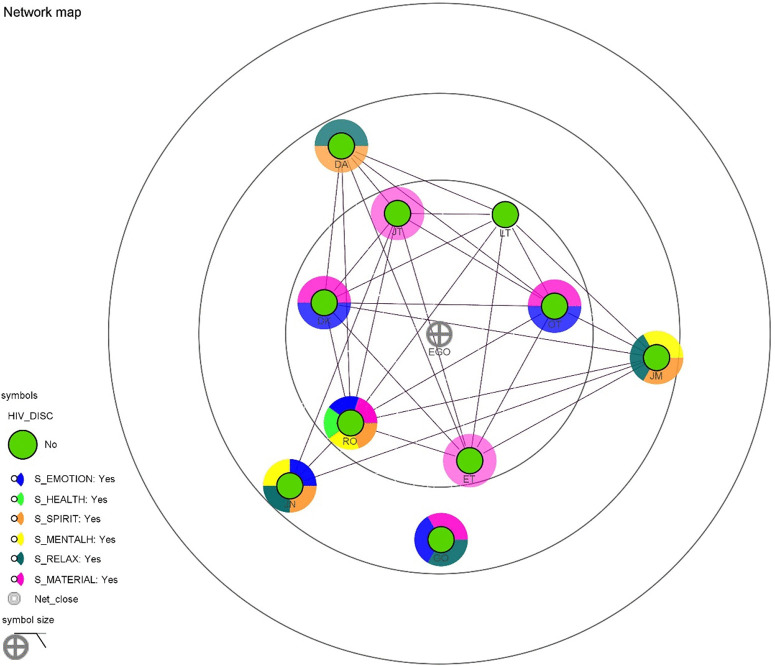
Mercy’s network map. Mercy has not disclosed to anyone in her network even though she receives support from nearly everyone; she does not want to worry her family and is not sure how others in her network will react, especially after having a bad experience disclosing to friends who are no longer in her network and not depicted on her map. Mercy prefers to handle her diagnosis privately, though she acknowledges she might disclose under future circumstances such as intensifying illness or major life transitions.

Mercy is a 32-year-old single woman who has not disclosed to anyone in her network map (Fig 7). She had previously disclosed to close friends, but faced mockery and judgement, which resulted in her cutting off these friends whom she did not name in her current network. This negative experience created internalized stigma for Mercy and shaped her hesitancy to further disclose to others. Even though she is supported by most people in her life, as seen in her map, she has not disclosed to her family because she does not feel that it is the “right time” to tell them. She does not want to “shock” them or “break their hearts.” Because she does not know how she will be treated by other network members, so she prefers to “just deal with it alone.” According to Mercy:

*You know, human beings are very funny, you might not know how somebody will treat you after they know life. This is how your status is…you can’t know how someone will treat you. You know I normally just prefer to just keep things to myself. Somebody’s reaction towards me may just change, especially people who don’t understand... they’ll start stigmatizing you.*

**Fig 8:**
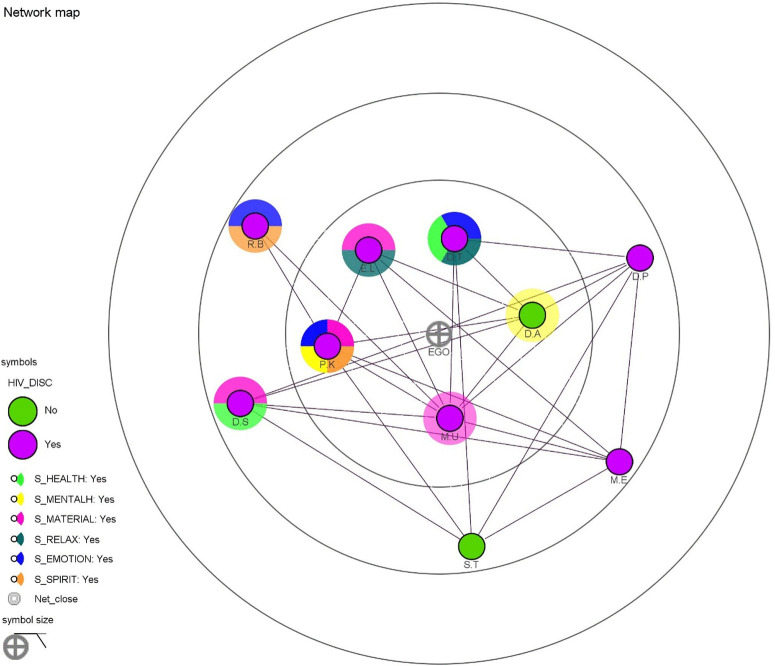
Grace’s network map. On the advice of healthcare workers (the plain purple dots in the middle circle), Grace disclosed to nearly all her network because she struggled with her mental health in the wake of her diagnosis. However, other alters to whom she disclosed are not on the map as they do not talk or interact anymore. While disclosing her HIV status has strengthened her relationships with the alters in the map, she remains hesitant to further disclose due to concerns about emotional impact and confidentiality.

Grace is a 40-year-old divorced woman who similarly experienced rejection after disclosing to friends, but her poor mental health pushed her to open up to others for support. She has disclosed to most of her network for support and spiritual guidance to help her navigate her diagnosis (Fig 8). Her inner circle includes her parents, a “spiritual parent,” and neighbor, and those in the middle circle are from a healthcare facility and friends, some of whom are also HIV + . She has not disclosed to a sibling (who is missing from her map) and has not disclosed to two people in her map, one friend on the edge of the middle circle because she does not trust him to keep a secret, and the other close family member whom she does not want to stress out with her diagnosis.

Notably, Grace talked about several others who do not appear in her network map; she initially disclosed to them, but after they learned of her status, “they don’t talk to me, and I don’t blame them.” She is working on becoming “strong enough” to disclose to everyone and not to internalize the shame, fear, and rejection she feels. Grace said the network members to whom she has disclosed have helped her navigate her mental health:

*Before I fully accepted my status, it really traumatized me, it affected me mentally, I started losing hope, I lost hope completely in living and I felt like killing myself. I attempted suicide so many times, but with time after counselling, talking to some few friends, I opened up with a few friends around me, some of them you could not think they are positive, so when they told me they are also in that category [HIV+] and they are living healthy, after some time I came out strong.*

Grace has actively engaged in her healthcare, including being prescribed antidepressants and counseled by healthcare workers (the purple dots in the middle circle, without support): “they told me you cannot make it if you keep it to yourself. It is going to affect you mentally, look for some people to talk to, so that is when I chose among my friends whom to talk to and I don’t regret it.” Since sharing, she has become closer to the people in her inner circle and received the support she needed. She is now doing much better, adhering to her medication and maintaining a healthy lifestyle.

## Discussion

HIV disclosure can play a critical role in HIV prevention, treatment, and care, leading to better physical and mental health outcomes for PLWHA. Studying disclosure through a social networks lens illustrates how sharing a diagnosis is a nuanced and personal decision made in relation to others. While the amount of disclosure among participants in our study varied, it was not the number of alters, but the quality of relationships and social support that mattered. We also found a complex relationship between disclosure, mental health, and stigma. Reflecting the disclosure process model, participants made calculated choices about disclosure to maximize their social support and improve mental health and wellbeing. On the flip side, when stigma and fear of social consequences prevented disclosure, mental health challenges were often exacerbated. For some participants, the alters missing in their current network maps indicate that relationships can indeed change after disclosure, which can inhibit decisions to disclose to others in the future. Our study suggests thinking beyond the clinical dimensions of HIV/AIDS to also prioritize programming that leverages social networks to support and encourage those newly diagnosed. We offer several key recommendations below.

Overall, disclosure was shaped by relationship closeness, trust, and support. These general patterns are visible in the participants’ network maps, in which the close proximity and multiple forms of support offered by alters tended to indicate disclosure (e.g., Fig 3, Victor) compared to alters in outer circles and alters with little support to offer, which often limited disclosure (e.g., Fig 2, Ruth). The qualitative data contextualize these relationships and help explain cases that would otherwise seem counterintuitive (e.g., Fig 7, Mercy), including situations where stigma prevented disclosure so as not to jeopardize important relationships. The network maps were a helpful prompt for reflection in the qualitative interviews, and in this analysis offer a snapshot of the disclosure event. In line with the disclosure process model, the qualitative data further explain how and why participants selectively disclosed to specific alters in their network and what resulted from sharing the diagnosis. In short, social relationships matter in HIV disclosure. Participants made selective decisions to tell alters who could support their wellbeing and adjustment to a new health condition.

Most people disclosed to their intimate partner, family members, and close friends. While we found that partners were largely supportive after disclosure, a new HIV diagnosis nonetheless raises difficult conversations that potentially threaten relationship trust [29]. Providing education and resources for intimate partners and supporting them through the testing process can strengthen disclosure, assist with difficult conversations, plan for the future, and disclose to children [22,30,39,40]. Besides partners, participants tended to share their status with supportive family members. However, they often selectively disclosed to mitigate family tensions or because they did not want to burden the people they cared about, suggesting that network interventions should consider familial dynamics [2]. Siblings emerged as particularly important sources of family support and represent an under-researched but promising set of social relationships in future intervention development [41].

Mental health and the need for psychological support emerged as a critical factor in deciding to disclose, particularly for women who were more likely than men to report a mental health issue in the survey and talk about experiencing stress, depression, and suicidal ideation in the qualitative interviews. These findings reflect the gendered burden and gaps in mental healthcare in SSA [42]. Those needing mental health support reached out to trusted alters in their networks, and the support, encouragement, acceptance, and prayers often improved participants’ mental health and emotional coping. Mental health support is needed upon diagnosis to avert crisis, as multiple women reported suicidal ideation and depression, but longer-term support for emotionally coping with a diagnosis should also be incorporated into interventions [42–44]. Healthcare providers also played an important role for some participants through encouraging disclosure and offering supportive advice when participants struggled with their mental health, suggesting that mental health training and crisis prevention should be incorporated into HIV counseling and testing programs [45,46].

However, given the critical shortage of mental health care in many lower- and middle-income countries, including Kenya, programming addressing the links between mental health and HIV care outside of the clinic should be an urgent priority. Peer support could prove helpful in this context [47–50], as participants who knew others in their networks who were HIV+ found their support and understanding helpful. Relatedly, participants sought spiritual support and turned to prayer to help them emotionally and psychologically through their diagnosis, suggesting that faith-based interventions could complement other forms of community-based mental healthcare to support PLWHA [51–53].

Stigma emerged as a critical factor shaping disclosure and negatively impacting participants’ health and wellbeing. As globally documented, forms of anticipated and internalized stigma prevented disclosure, sometimes even within close relationships [10,13,54]. A recent national survey in Kenya found that stigma has far-ranging effects, including reluctance to be tested for HIV, fear of negative reactions from others, and lack of engagement in HIV care [27]. Stigma and interrelated gender inequities are particularly salient for Kenyan women navigating disclosure decisions in the context of their structural vulnerability and poor mental health [19].

The intersection of social relationships, mental health, and stigma is critical in understanding how the disclosure process model operates in this context. Selective disclosure to close, trusted alters typically helped participants navigate their diagnosis with the support and care they received after disclosure. This support was particularly important for those struggling with their mental health in the wake of an HIV diagnosis. On the other hand, concealment of one’s status due to stigma often exacerbated distress and prevented further disclosure with others. Participants’ fears of the social consequences of disclosure should be acknowledged: these concerns are valid, especially considering that some participants’ networks changed in the wake of their HIV disclosure. In these cases, alters were *missing* from participants’ current network maps after they disclosed. These participants not only lost forms of support, they often internalized the stigma and discrimination they experienced as a result of sharing their HIV diagnosis. These socially harmful experiences made participants hesitant to disclose to others for fear of being mistreated and publicly outed and shamed. PLWHA should not shoulder the entire burden of disclosure; the broader social conditions that enable the HIV epidemic to persist through stigma, culturally conditioned silence, and acts of discrimination must be addressed. The multifaceted impact of HIV-related stigma on disclosure decisions suggests that HIV programs should address stigma at multiple levels, including the societal level through awareness campaigns, education, and community engagement [55]. Such programs should involve PLWHA and could emphasize that everyone deserves respect and care and can live healthy lives with social support. Multiple participants found support from other PLWHA in their network particularly important, as these social relationships fostered open conversation to overcome stigma and cultivate support. Involving other PLWHA is key to social network interventions to offer hope and change harmful social conditions.

Our study is limited by its cross-sectional design, preventing us from making causal inferences. Our mental health data are self-reported from the CDQ and thus not clinical diagnoses. Our results should also be interpreted with caution due to the biases inherent in data collection, including social desirability and recall bias. We also closed our recruitment in the networks study earlier than anticipated due to the global pandemic, which reduced our sample and may have impacted our results. However, study strengths include its mixed methods design, including incorporating the visual mapping of social network survey data. The social network maps contribute an additional layer of information, illustrating variation in disclosure patterns, while the qualitative data revealed the complex, overlapping factors that shape disclosure decisions not as one-time conversation, but as a process in which PLWHA must decide when and why it is advantageous to share their diagnosis.

Overall, our study suggests that social networks could be leveraged in interventions to support the health and wellbeing of PLWHA. Programs should consider active involvement of the partners, family members, friends, and peers of newly diagnosed individuals in interventions. Campaigns to reduce community-level stigma around HIV are necessary to foster a more supportive environment to increase disclosure. The involvement of PLWHA and supportive healthcare workers could offer critical support in such efforts. All such programming should take a holistic approach that addresses not only the clinical aspects of HIV care but also the social, psychological, and spiritual dimensions of a new diagnosis and how this impacts social relationships and wellbeing.

## Supporting Information

S1 Checklist Inclusivity in global research. (DOCX)
